# Country of Birth, Race, Ethnicity, and Prenatal Depression

**DOI:** 10.1001/jamanetworkopen.2025.31844

**Published:** 2025-09-15

**Authors:** Kendria Kelly-Taylor, Sara Aghaee, Joshua Nugent, Nina Oberman, Ai Kubo, Elaine Kurtovich, Charles P. Quesenberry, Ayesha C. Sujan, Kathryn Erickson-Ridout, Mibhali M. Bhalala, Lyndsay A. Avalos

**Affiliations:** 1 Division of Research, Kaiser Permanente Northern California, Pleasanton; 2 Stanford University School of Medicine, Stanford, California; 3 The Permanente Medical Group, Kaiser Permanente Northern California, Oakland; 4 Kaiser Permanente Redwood City Medical Center, Redwood City, California; 5 Department of Health Systems Science, Kaiser Permanente Bernard J. Tyson School of Medicine, Pasadena, California

## Abstract

**Importance:**

Non–US-born pregnant individuals have demonstrated better perinatal outcomes compared with their US-born counterparts, yet limited literature has explored this association among mental health conditions in pregnancy and across racial and ethnic groups.

**Objective:**

To examine the differences in prenatal depression diagnosis and moderate to severe depression symptoms between non–US-born and US-born individuals across racial and ethnic subgroups.

**Design, Setting, and Participants:**

Cross-sectional study of members of Kaiser Permanente Northern California (KPNC), an integrated health care delivery system, who attended at least 1 prenatal care visit and delivered a live birth between January 1, 2013, and December 31, 2019. Data were analyzed from September 2023 to January 2024.

**Exposures:**

Self-reported race, ethnicity, and country of birth. Country of birth was used to define maternal nativity (US-born vs non–US-born).

**Main Outcomes and Measures:**

Prenatal depression diagnosis (PDD) defined by *International Classification of Diseases, Ninth Revision *and* Tenth Revision* codes and moderate to severe depression symptoms defined by self-reported Patient Health Questionnaire-9 (PHQ-9) scores of 10 or greater documented in the KPNC electronic health records (EHR) between the first day of the last menstrual period to the day before live birth.

**Results:**

Among the 252 171 participants (168 605 [66.7%] US-born and 83 566 [33.1%] non-US-born), adjusted models showed non–US-born pregnant individuals had an equivalent or significantly lower risk of PDD compared with their US-born counterparts within racial and ethnic subgroups. Non–US-born individuals presented a higher risk of moderate to severe depression symptoms compared with US-born individuals among certain Hispanic (eg, adjusted relative risk [aRR] for other Hispanic individuals, 1.30; 95% CI, 1.01-1.67), and Asian (eg, aRR for Japanese individuals, 3.62; 95% CI, 2.08-6.30) subgroups as well as among White pregnant individuals (aRR, 1.17; 95% CI, 1.10-1.25). Non–US-born Black pregnant individuals presented lower risk of PDD (aRR, 0.30; 95% CI, 0.25-0.36) and moderate to severe depression symptoms (aRR, 0.75; 95% CI, 0.65-0.86) compared with US-born Black individuals.

**Conclusions and Relevance:**

Across racial and ethnic groups, PDD and moderate to severe depression symptoms varied by maternal nativity in this cross-sectional study. The observed advantage among non–US-born individuals across other maternal and neonatal outcomes may not uniformly apply to prenatal mental health conditions when race and ethnicity are considered. Future research should explore sociocultural factors that may influence this association.

## Key Points

**Question:**

Do the risks of prenatal depression and moderate to severe depression symptoms differ among US-born and non–US-born pregnant individuals across racial and ethnic subgroups?

**Findings:**

In this cross-sectional study of 252 171 pregnant individuals, non–US-born individuals had a lower risk of prenatal depression diagnosis but a higher risk of moderate to severe depression symptoms, with variations by race and ethnicity.

**Meaning:**

These findings suggest future research should consider nativity as an important factor associated with disparities in prenatal depression; mechanisms that prioritize sociocultural factors should be explored to contextualize the intersectional association between nativity, race, ethnicity, and prenatal mental health.

## Teaser

This cross-sectional study examines the differences in prenatal depression diagnosis and moderate to severe depression symptoms between non–US-born and US-born individuals across racial and ethnic subgroups.

## Introduction

Racial and ethnic disparities in perinatal outcomes continue to be of public health concern. Of increasing interest is the role of nativity (US-born vs non–US-born) and how this may contribute to disparities, particularly as immigrant populations of racial and ethnic minorities (eg, Hispanic) are the fastest-growing in the US.^1^ Most research has focused on adverse birth outcomes like preterm birth and low birth weight, with many studies suggesting that non–US-born individuals tend to have better outcomes than their US-born counterparts.^2,3,4^ Traditionally, the healthy immigrant effect—broadly defined as the phenomenon that non–US-born individuals have better health outcomes compared with their US-born counterparts despite lower socioeconomic status^5,6^—has been hypothesized to explain these differences.

However, more recent research has highlighted the heterogeneity in perinatal outcomes among immigrant populations with differences emerging by race, ethnicity, and country of birth.^7^ Despite growing attention to this variation, less is known about the association between maternal nativity and mental health conditions in pregnancy and how this varies across racial and ethnic groups.

Prenatal depression, a leading mental health condition that affects between 10% to 30% of pregnant individuals, is associated with adverse maternal (eg, postpartum depression) and child (eg, developmental delays) health outcomes, with potential multigenerational effects.^8,9^ Research suggests that Black, Asian, and Hispanic pregnant individuals are at a heightened risk of prenatal depression compared with White individuals,^8,10^ though findings vary.^11^ The difference in study findings could be attributed to within-group differences, such as maternal nativity.^12^ The relationship between maternal nativity and prenatal depression, especially across racial and ethnic groups, remains underexplored. Findings from existing research are mixed. For example, 1 study found that non–US-born individuals had a lower risk of prenatal depression compared with US-born individuals, though racial and ethnic differences were not explored.^13^ In contrast, another study discovered that non–US-born Hispanic pregnant individuals who resided in the US for over 20 years had higher odds of prenatal depression compared with their US-born counterparts.^14^

Nativity often serves as a proxy for health-related factors such as acculturation, immigration, and cultural norms.^15^ Pivotal to one’s identity and socialization within US society, nativity is an important indicator to one’s health and thus should be explored across multiple health conditions. Furthermore, studies suggest that nativity and related factors (eg, acculturation) may operate differently depending on the racial and ethnic group based on differences in structural (eg, polices that advantage one racial and ethnic group and disadvantage another) and cultural (eg, mental health stigma) factors.^16^ The relationship between nativity, race and ethnicity, and mental health conditions is nuanced. An exploration of this relationship could assist in the development and implementation of culturally relevant interventions aimed at reducing the burden of mental health conditions, especially during pregnancy among diverse populations. To our knowledge, this is the first study to explore the differences in prenatal depression diagnosis and moderate to severe depression symptoms among US-born and non–US-born individuals across racial and ethnic subgroups in a large diverse cohort of pregnant individuals who received universal screening for depression during pregnancy.

## Methods

### Study Population

A retrospective cross-sectional study design was conducted among Kaiser Permanente Northern California (KPNC) members who had a singleton live birth from January 1, 2013, to December 31, 2019; were between ages 15 to 45 years at delivery; attended at least 1 KPNC prenatal care visit; and had self-reported information on race, ethnicity, and country of birth (see eFigure in Supplement 1 for study sample criteria). This study followed the Strengthening the Reporting of Observational Studies in Epidemiology (STROBE) reporting guideline for cross-sectional studies and was approved by both the KPNC institutional review board and California state institutional review board. The requirement of informed consent was waived because data were deidentified. Study procedures meet Health Insurance Portability and Accountability Act requirements and 42 CFR part 2 regarding medical records. Upon enrolling in the KPNC health plan, all members are informed that their data may be used for research.

### Exposure

Maternal nativity was defined by self-reported country of birth and categorized into US-born and non–US-born for each racial and ethnic group. Non–US-born individuals included those born outside the continental US, Alaska, and Hawaii. Race, ethnicity, and country of birth were self-reported and ascertained from California state birth records (primary) and KPNC electronic health records (EHR) and relevant databases (secondary). Race and ethnicity were first categorized into larger groups including Asian, Hispanic, non-Hispanic White (hereafter referred to as White), and non-Hispanic Black (hereafter referred to as Black), then further categorized into subgroups. Individuals who identified as multiracial or did not have a specified origin (ie, country of birth) were excluded. Nineteen racial and ethnic groups were identified. These groups are as follows: Asian Indian, Black, Cambodian, Central or South American, Chinese, Filipina, Hmong, Japanese, Korean, Laotian, Mexican, Native Hawaiian or Pacific Islander, other Hispanic, other South Asian, other Southeast Asian, Puerto Rican, Thai, Vietnamese, and White.

### Outcomes

Prenatal depression diagnosis (PDD) was defined by *International Classification of Diseases, Ninth Revision *and *Tenth Revision* (see eTable 1 in Supplement 1 for codes). Moderate to severe depression symptoms were defined by a Patient Health Questionnaire-9 (PHQ-9) score of 10 or greater. PHQ-9 is a validated self-reported measure of depression severity^17^ and administered twice in pregnancy (at the first prenatal care visit and at 24-28 gestational weeks) as part of KPNC’s universal prenatal care screening program.^18,19^ For analysis, the highest PHQ-9 score was used. A PHQ-9 score of 10 or greater aligns with the clinical threshold for depression diagnosis.^17^ According to KPNC clinical guidelines, when a patient presents a PHQ-9 score of 10 or greater, clinicians engage in further discussions about symptoms and relevant medical history, using their clinical judgement to determine a formal diagnosis. More details regarding KPNC’s guidelines can be found elsewhere.^18^ Outcomes were documented in KPNC EHRs between the first day of the last menstrual period through the day before a live birth.

### Statistical Analysis

The relative risk of diagnosis and moderate to severe depression symptoms for non–US-born individuals compared with US-born pregnant individuals (the reference category) and the associated 95% CIs were estimated using a series of modified Poisson regression models^20^ among each identified racial and ethnic group. Models were adjusted for confounders identified a priori (maternal age, education, parity, neighborhood deprivation index, and delivery year). Neighborhood deprivation index served as a proxy for individual-level socioeconomic status variables, such as income, and as an indicator of broader structural and economic disadvantages not captured in our dataset. Adjusting for this variable in our model helps isolate the role of nativity while holding potentially racialized experiences more constant. All analyses were conducted using SAS version 9.4 (SAS Institute), and 2-sided *P* < .05 was considered statistically significant. Data were analyzed from September 2023 to January 2024.

## Results

Among the study sample of 252 171 individuals, approximately two-thirds were US-born (168 605 individuals [66.7%]) while one-third were non–US-born (83 566 individuals [33.1%]). A higher proportion of non–US-born pregnant individuals were older than 30 years of age (55 421 individuals [63.3%] vs 89 469 individuals [53.1%]), were multiparous (49 018 individuals [58.7%] vs 92 755 individuals [55.0%]), had a college degree or higher (50 991 individuals [48.7%] vs 83 825 individuals [40.9%]), and did not use Medicaid (77 970 individuals [93.3%] vs 149 566 individuals [88.7%]) compared with US-born pregnant individuals. Among the study population, the prevalence of prenatal depression diagnosis was 15.6% (39 258 individuals), with a lower prevalence observed among non-US-born individuals at 8.4% (7018 individuals), compared with 19.1% (32 240 individuals) among US-born individuals. Among non–US-born and US-born pregnant individuals, the prevalence of moderate to severe depression symptoms were similar, with non–US-born individuals presenting a slightly higher prevalence (10.3% vs 9.7%) (Table).

**Table.** Descriptive Characteristics of Kaiser Permanente Northern California (KPNC) Pregnant Individuals Between January 1, 2013, to December 31, 2019

Characteristic	Participants, No. (%)		
	Total sample (N = 252 171)	US-born (n = 168 605)	Non–US-born (n = 83 566)
Race and ethnicity			
Asian and Pacific Islander			
Asian Indian	15 789 (6.3)	1365 (0.8)	14 424 (17.3)
Cambodian	1104 (0.4)	471 (0.3)	633 (0.8)
Chinese	15 394 (6.1)	4802 (2.8)	10 592 (12.7)
Filipina	14 889 (5.9)	5680 (3.4)	9209 (11.0)
Hawaiian/Pacific Islander	1876 (0.7)	955 (0.6)	921 (1.1)
Hmong	2938 (1.2)	1960 (1.2)	978 (1.2)
Japanese	1244 (0.5)	534 (0.3)	710 (0.9)
Korean	2224 (0.9)	754 (0.5)	1470 (1.8)
Laotian	670 (0.3)	330 (0.2)	340 (0.4)
Thai	572 (0.2)	83 (0.1)	489 (0.6)
Vietnamese	6004 (2.4)	1617 (1.0)	4387 (5.3)
Other South Asian^a^	1493 (0.6)	187 (0.1)	1306 (1.6)
Other Southeast Asian^b^	508 (0.2)	110 (0.1)	398 (0.5)
Hispanic			
Central/South American	8493 (3.4)	3504 (2.1)	4989 (6.0)
Mexican	56 086 (22.2)	38 802 (23.0)	17 284 (20.7)
Puerto Rican	1507 (0.6)	1375 (0.8)	132 (0.2)
Other Hispanic^c^	7323 (2.9)	6755 (4.0)	568 (0.7)
Black	15 574 (6.2)	13 473 (8.0)	2101 (2.5)
White	98 483 (39.1)	85 848 (50.9)	12 635 (15.1)
Prenatal depression diagnosis^d^			
Yes	39 258 (15.6)	32 240 (19.1)	7018 (8.4)
No	212 913 (84.4)	136 365 (80.9)	76 548 (91.6)
Depression symptom severity^e^			
None to mild, PHQ-9 score 0-9^f^	202 489 (80.3)	136 318 (80.9)	66 171 (79.2)
Moderate to severe, PHQ-9 score ≥10	25 856 (10.3)	16 283 (9.7)	8573 (10.3)
Missing	24 826 (9.9)	16 004 (9.4)	8822 (10.6)
Maternal age, y			
15-24	37 994 (15.1)	31 454 (18.7)	6540 (7.8)
25-29	69 287 (27.5)	47 682 (28.3)	21 605 (25.9)
30-34	90 678 (36.0)	57 957 (34.4)	32 721 (36.1)
35-45	54 212 (21.5)	31 512 (18.7)	22 700 (27.2)
Education			
Less than high school	7357 (2.9)	3646 (2.2)	3711 (4.4)
High school graduate/GED	39 059 (15.5)	27 490 (16.3)	11 569 (13.8)
Some college	70 939 (28.1)	53 644 (31.8)	17 295 (20.7)
Bachelor’s degree	67 149 (26.6)	43 567 (25.8)	23 582 (28.2)
Postgraduate	42 534 (16.9)	25 440 (15.1)	17 094 (20.5)
Missing	25 133 (10.0)	14 818 (8.8)	10 315 (12.3)
Parity			
0	110 398 (43.8)	75 850 (45.0)	34 548 (41.3)
1	87 544 (34.7)	57 198 (33.9)	30 346 (36.3)
≥2	52 766 (20.9)	34 570 (20.5)	18 196 (21.8)
Missing	1463 (0.6)	987 (0.6)	476 (0.6)
Medicaid			
No	227 536 (90.3)	149 566 (88.7)	77 970 (93.3)
Yes	24 635 (9.8)	19 039 (11.3)	5596 (6.7)
Neighborhood deprivation index quartile^g^			
1 (least deprived)	62 661 (24.9)	38 949 (23.1)	23 712 (28.4)
2	63 158 (25.1)	43 333 (25.7)	19 825 (23.7)
3	63 010 (24.8)	43 417 (25.8)	19 593 (23.5)
4 (most deprived)	62 501 (24.8)	42 381 (25.1)	20 120 (24.1)
Missing	841 (0.3)	525 (0.3)	316 (0.4)
Delivery year			
2013	32 648 (13.0)	21 728 (12.9)	10 920 (13.1)
2014	34 515 (13.7)	23 089 (13.7)	11 426 (13.7)
2015	36 307 (14.4)	24 233 (14.4)	12 074 (14.5)
2016	37 698 (15.0)	25 089 (14.9)	12 609 (15.1)
2017	37 900 (15.0)	25 162 (14.9)	12 738 (15.2)
2018	35 727 (14.2)	23 871 (14.2)	11 856 (14.2)
2019	37.376 (14.8)	25 433 (15.1)	11 943 (14.3)

^a^ Other South Asian includes individuals self-identified as Pakistani, Nepalese, Sri Lankan, and Bangladeshi/Bengali.

^b^ Other Southeast Asian includes Malaysian, Indonesian, Singaporean, and Burmese.

^c^ Other Hispanic includes additional countries where the primary spoken language is Spanish or individuals from multiple Latin American regions.

^d^ Prenatal depression diagnosis was defined by *International Classification of Diseases, Ninth Revision* and *Tenth Revision* codes and documented between first day of the last menstrual period through day before a live birth.

^e^ Depression severity was measured by Patient Health Questionnaire-9 (PHQ-9) and documented between the first day of the last menstrual period through the day before live birth. PHQ-9 is a validated self-reported depression screener and administered to pregnant individuals as part of KPNC’s standard prenatal care twice during pregnancy (first prenatal visit and 24-28 weeks at the glucola visit).^18^

^f^ None to mild depression symptoms was defined as PHQ-9 score 0 to 9, and moderate to severe depression symptoms was defined as PHQ-9 score of 10 or greater. A PHQ-9 score of 10 or greater is consistent with the clinical threshold for a depression diagnosis.

^g^ Neighborhood deprivation was defined by the neighborhood deprivation index.

**Figure 1.**
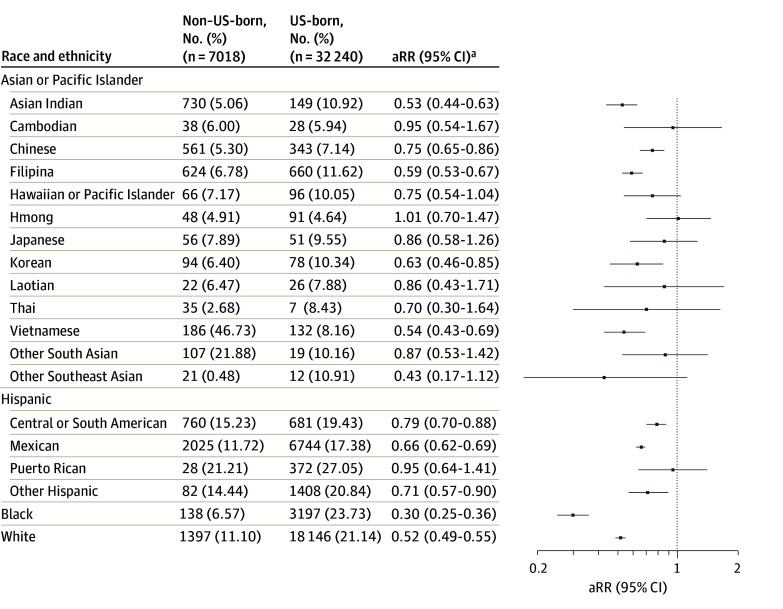
Relative Risk of Prenatal Depression Diagnosis of Non−US-Born Compared With US-Born Pregnant Individuals by Race and Ethnicity ^a^Adjusted for maternal age, education, parity, delivery year, and NDI. aRR indicates adjusted adjusted relative risk.

Non–US-born pregnant individuals had significantly lower or equivalent risks of prenatal depression diagnosis (PDD) compared with US-born counterparts across racial and ethnic subgroups. The lowest risk was observed among non–US-born Black individuals whose risk of prenatal depression diagnosis was 70% lower compared with US-born Black individuals (adjusted relative risk [aRR], 0.30; 95% CI, 0.25-0.36). Likewise, a lower risk of PPD was observed among non–US-born compared with US-born individuals across Asian subgroups, including Asian Indian (aRR, 0.53; 95% CI, 0.44-0.63), Chinese (aRR, 0.75; 95% CI, 0.65-086), Filipina (aRR, 059; 95% CI, 0.53-0.67), Korean (aRR, 0.63; 95% CI, 0.46-0.85), and Vietnamese (aRR, 0.54; 95% CI, 0.43-0.69) individuals; Hispanic subgroups such as Central or South American (aRR, 0.79; 95% CI, 0.70-0.88), Mexican (aRR, 0.66; 95% CI, 0.62-0.69), and other Hispanic (aRR, 0.71; 95% CI, 0.57-0.90) individuals; and White pregnant individuals (aRR, 0.52; 95% CI, 0.49-0.55) (Figure 1).

**Figure 2.**
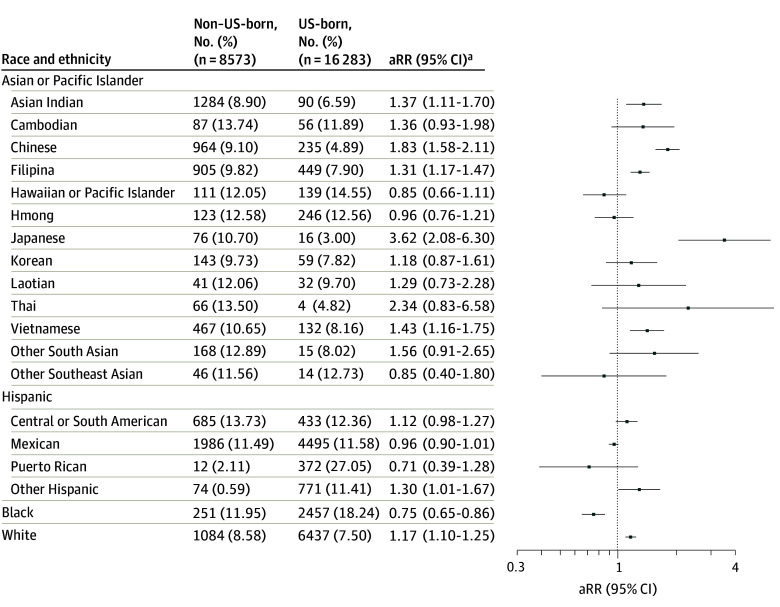
Relative Risk of Moderate to Severe Depression Symptoms of Non−US-Born Compared With US-Born Pregnant Individuals by Race and Ethnicity ^a^Adjusted for maternal age, education, parity, delivery year, and NDI. aRR indicates adjusted adjusted relative risk.

Non–US-born Black pregnant individuals had a significantly lower risk of moderate to severe depression symptoms compared with US-born Black individuals (aRR, 0.75; 95% CI, 0.65-0.86). However, in other racial and ethnic groups, non–US-born individuals showed an equivalent or higher risk. Among Asian subgroups, Asian Indian, Chinese, Filipina, Japanese, and Vietnamese non–US-born (vs US-born) pregnant individuals had a higher risk of moderate to severe depression symptoms. The highest risk was observed among Japanese non–US-born individuals (aRR, 3.62; 95% CI, 2.08-6.30). Among Hispanic subgroups, Central or South American (aRR, 1.12; 95% CI, 0.98-1.27) and Other Hispanic (aRR, 1.30; 95% CI, 1.01-1.67) also had a slightly higher risk among non–US-born individuals. For White pregnant individuals, non–US-born individuals had 17% higher risk of moderate to severe depression symptoms compared with US born individuals (aRR, 1.17; 95% CI, 1.10-1.25) (Figure 2).

## Discussion

The risk of prenatal depression diagnosis and moderate to severe depression symptoms among non–US-born individuals compared with US-born individuals differed across racial and ethnic groups in our population. Our study highlights the importance of exploring the association between nativity and depression among disaggregated racial and ethnic subgroups, as our study shows variation in risk within Asian and Hispanic subgroups by nativity. Previous studies have documented that the risk of depression differs by Asian and Hispanic subgroups acknowledging the heterogeneity among these groups.^21,22^ Exploring the role of nativity on the risk of depression expands our knowledge of these existing racial and ethnic subgroup differences by adding an additional layer of identity that could assist in our understanding of prenatal mental health disparities.

Moreover, our findings demonstrate that non–US-born pregnant individuals had an equivalent or lower risk of prenatal depression diagnosis, yet an equivalent or higher risk of moderate to severe depression symptoms compared with their US-born counterparts across most racial and ethnic subgroups. The observed higher risk of depression symptoms yet lower risk of depression diagnosis could partly be due to language barriers, lack of cultural competency in clinician settings, or culturally based stigma, all of which coincide with factors associated with mental health treatment engagement.^23^ Additionally, our findings may point to a nuanced area of investigation related to clinical underdiagnosis. A recent study^24^ among pregnant individuals at KPNC found that the risk of clinical underdiagnosis, defined as having a PHQ-9 score of 10 or higher without a corresponding depression diagnosis, was significantly higher among Asian and Hispanic subgroups (excluding Puerto Rican) and Black individuals compared with White individuals. Although that study did not account for nativity, its findings may be relevant here, suggesting that clinical underdiagnosis among immigrant populations could partially explain the observed discrepancies between moderate to severe depressive symptoms and clinical diagnoses. However, further research is needed to explore this possibility. Interestingly, non–US-born Black pregnant individuals had significantly lower risk of both depression diagnosis and moderate to severe depression symptoms compared with their US-born counterparts. This may be due to country-of-origin differences; prior studies have found health variations among Black immigrants based on origin, with more favorable perinatal outcomes observed among those from Sub-Saharan Africa than the Caribbean.^25^ Black immigrants from Caribbean countries often migrate at a younger age and live longer in the US, potentially increasing exposure to racial discrimination and its subsequent adverse health consequences.^16,25^ It is possible that our study includes a higher proportion of non–US-born-Black pregnant individuals who recently immigrated from Sub-Saharan Africa, which may be associated with lower exposure to social and systemic discrimination. This, in turn, could contribute to a reduced risk of both depression diagnosis and moderate to severe depression symptoms. However, future research is needed to support this hypothesis in our study.

Additionally, across all racial and ethnic groups, our findings could be related to uncaptured measures of acculturation such as length of residency and age at migration,^26^ differences in cultural norms and perception of mental health stigma, medical distrust,^27^ and immigrant status.^28^ Moreover, unmeasured factors such as immigration-related policies and heightened fear of encountering law enforcement could potentially contribute to the heightened severity of depression observed among immigrant populations.^29^ These factors may play a critical role in the observed association, especially as non–US-born pregnant individuals in our sample had higher socioeconomic status (eg, lower proportion of Medicaid utilization) compared with their US-born counterparts. This suggests that our findings expand beyond the healthy immigrant effect and other factors should be considered. Future research should explore how the interplay of these variables relates to the differences between non–US-born and US-born risk of prenatal depression diagnosis and moderate to severe depression symptoms among racial and ethnic groups.

### Limitations

This study had several limitations. Generalizability of our study findings may be limited for uninsured and potentially publicly insured immigrant pregnant individuals, as the proportion of immigrants on Medicaid in our sample (6.7%) is lower than that of the broader US immigrant population eligible for Medicaid (approximately 19%).^30^ However, with membership covering about 40% of Northern California, the KPNC population reflects the demographics of the surrounding area.^31^ This study did not consider individual factors like age at migration, length of residence, immigration or visa status, employment, or systemic barriers like residential segregation or immigration policies. These factors could further explain differences in prenatal depression risk between US-born and non–US-born pregnant individuals. This study did not explore pregnant individuals who self-identified as American Indian or Alaskan Native due to a small sample size of those who identified as non–US-born pregnant individuals.

Despite these limitations, this study offers several important strengths. This is the first study that has explored the differences in prenatal depression diagnosis and symptom severity by maternal nativity among a large, diverse population of pregnant individuals with self-reported race, ethnicity, and country of birth. Self-reported depression symptoms were captured via the PHQ-9, a measure of depression severity with high sensitivity and specificity among obstetric patients.^17^ In addition, clinical diagnoses and self-reported depression symptoms were captured during KPNC prenatal screenings. This can assist in reducing recall and misclassification bias by providing a population screen for mental health during pregnancy.

## Conclusion

The study found notable differences in the risk of depression diagnosis and moderate to severe depression symptoms among US-born and non–US-born pregnant individuals across racial and ethnic groups. The results showcase the importance of considering maternal nativity, along with race and ethnicity, when examining disparities in mental health conditions in pregnancy as differences in social identities can be associated with risk. Future research should explore additional factors to better understand the intersection of nativity, race and ethnicity, and mental health in pregnancy, aiding the development of culturally appropriate interventions to reduce the burden of adverse prenatal mental health conditions.

## Notes

Supplement 1. **eFigure. **Flow Diagram of Study Sample **eTable. ***International Classification Of Diseases, Ninth Revision, Clinical Modification (ICD-9)* and the *International Statistical Classification of Diseases, Tenth Revision, Clinical Modification (ICD-10)* Codes for Depression

Supplement 2.
Data Sharing Statement
