# Supplementary material for: Country of Birth, Race, Ethnicity, and Prenatal Depression
Source: JAMA Netw Open. 2025 Sep 15;8(9):e2531844. doi: 10.1001/jamanetworkopen.2025.31844 (PMC12439054; doi:10.1001/jamanetworkopen.2025.31844)
Supplement: Supplement 1. — eFigure. Flow Diagram of Study Sample eTable. International Classification Of Diseases, Ninth Revision, Clinical Modification (ICD-9) and the International Statistical Classification of Diseases, Tenth Revision, Clinical Modification (ICD-10) Codes for Depression [file jamanetwopen-e2531844-s001.pdf]

Supplemental Online Content

Kelly-Taylor K, Aghaee S, Nugent J, et al. Country of birth, race, ethnicity, and prenatal depression. *JAMA Netw Open*. 2025;8(9):e2531844. doi:10.1001/jamanetworkopen.2025.31844

eFigure. Flow Diagram of Study Sample.
eTable. *International Classification of Diseases, Ninth Revision, Clinical Modification (ICD-9)* and the *International Statistical Classification of Diseases, Tenth Revision, Clinical Modification (ICD-10)* Codes for Depression

This supplemental material has been provided by the authors to give readers additional information about their work.

© 2025 Kelly-Taylor K et al. *JAMA Network Open*.

eFigure 1. Flow Diagram of Study Sample

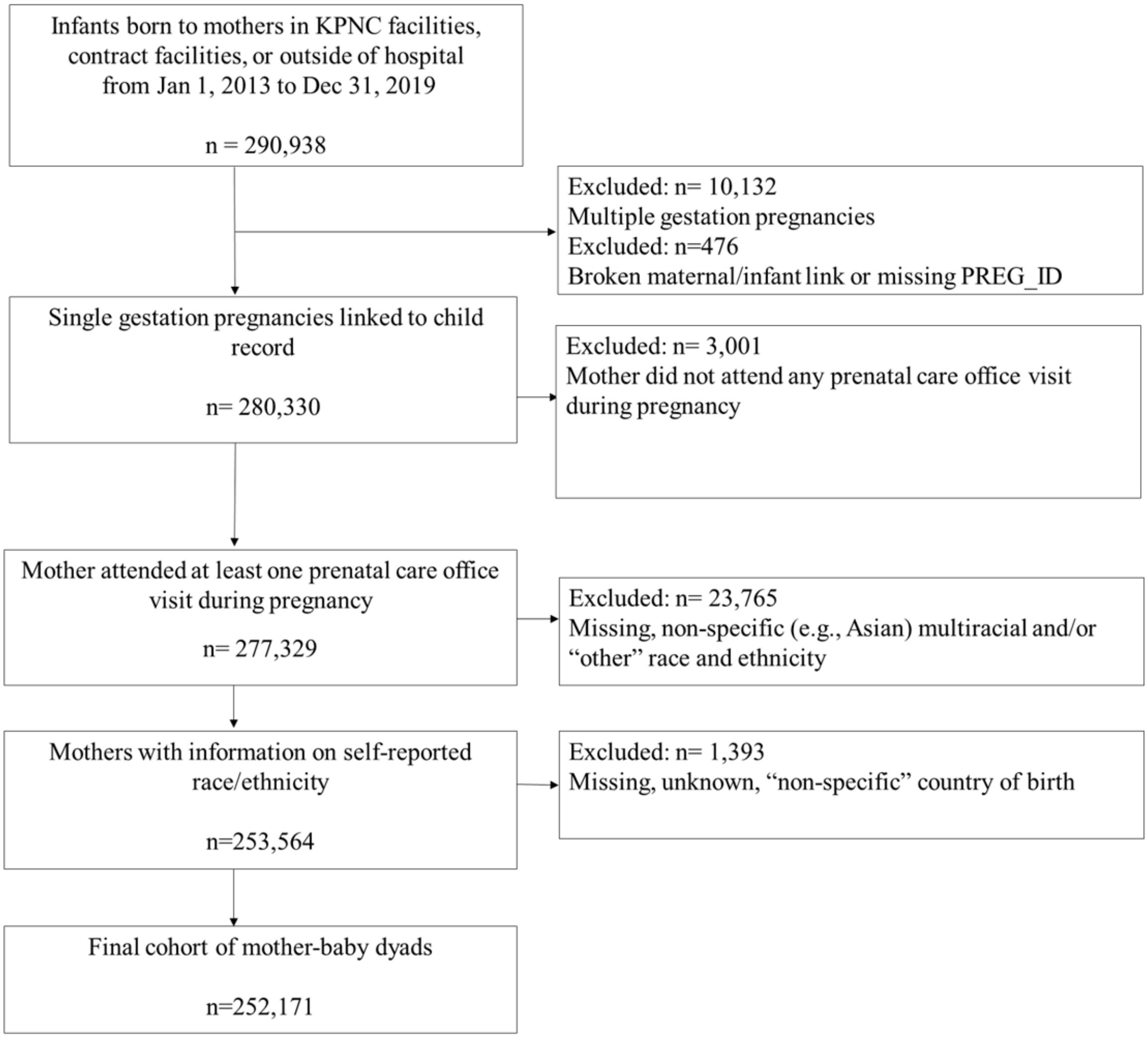

© 2025 Kelly-Taylor K et al. *JAMA Network Open*.

**eTable.** ***International Classification of Diseases, ninth revision, Clinical Modification (ICD-9)*** **and the *International Statistical Classification of Diseases, tenth revision, Clinical Modification (ICD-10)* Codes for Depression**	
**ICD-9**	296.2, 296.21, 296.22, 296.23, 296.24, 296.25, 296.3, 296.31, 296.32, 296.33, 296.34, 296.35, 296.82, 298, 300.4, 309.1, 309.28, 311, 648.4, 648.41, 648.42, 648.43, 648.44
**ICD-10**	F32.0, F32.1, F32.2, F32.3, F32.4, F32.8, F32.81, F32.89, F32.9, F33.0, F33.1, F33.2, F33.3, F33.41, F33.8, F33.9, F34.1, F43.21, F43.23, F53.0, O99.34, O99.340, O99.341, O99.342, O99.343, O99.344, O99.345, O90.6

© 2025 Kelly-Taylor K et al. *JAMA Network Open*.
