# Supplementary material for: Country of Birth, Race, Ethnicity, and Prenatal Depression
Source: JAMA Netw Open. 2025 Sep 15;8(9):e2531844. doi: 10.1001/jamanetworkopen.2025.31844 (PMC12439054; doi:10.1001/jamanetworkopen.2025.31844)
Supplement: Supplement 2. — Data Sharing Statement [file jamanetwopen-e2531844-s002.pdf]

Data Sharing Statement

Kelly-Taylor. Country of Birth, Race, Ethnicity, and Prenatal Depression. *JAMA Netw Open*. Published September 15, 2025. doi:10.1001/jamanetworkopen.2025.31844

Data
Data available: No
